# COVID-19 Vaccination in Patients with Pulmonary Arterial Hypertension and Chronic Thromboembolic Pulmonary Hypertension: Safety Profile and Reasons for Opting against Vaccination

**DOI:** 10.3390/vaccines9121395

**Published:** 2021-11-25

**Authors:** Maria Wieteska-Miłek, Sebastian Szmit, Michał Florczyk, Beata Kuśmierczyk-Droszcz, Robert Ryczek, Marcin Kurzyna

**Affiliations:** 1 Department of Pulmonary Circulation, Thromboembolic Diseases and Cardiology, Centre of Postgraduate Medical Education, European Health Center, 05-400 Otwock, Poland; sszmit@cmkp.edu.pl (S.S.); michal_florczyk@wp.pl (M.F.); mkurzyna@cmkp.edu.pl (M.K.); 2 Department of Congenital Heart Disease, National Institute of Cardiology, 04-628 Warsaw, Poland; bkusmier@gmail.com; 3 Department of Cardiology and Internal Diseases, Military Institute of Medicine, 04-349 Warsaw, Poland; raryczek@gmail.com

**Keywords:** pulmonary arterial hypertension, chronic thromboembolic pulmonary hypertension, COVID-19 vaccine, vaccination, post-vaccination symptoms, safety

## Abstract

The incidence of COVID-19 infection in patients with pulmonary arterial hypertension (PAH) and chronic thromboembolic pulmonary hypertension (CTEPH) is similar to that in the general population, but the mortality rate is much higher. COVID-19 vaccination is strongly recommended for PAH/CTEPH patients. The aim of our cross-sectional study was to identify reasons why PAH/CTEPH patients refused vaccination against COVID-19. Moreover, we assessed the safety profile of approved COVID-19 vaccines in PAH/CTEPH patients. We examined 261 patients (164 PAH patients and 97CTEPH patients) with a median age of 60 (18–92) years, 62% of which were female. Sixty-one patients (23%) refused to be vaccinated. The main reason for unwillingness to be vaccinated was anxiety about adverse events (AEs, 61%). Age and fear of COVID-19 in the univariate analysis and age ≥60 years in the multivariate regression analysis were factors that impacted willingness to be vaccinated (OR = 2.5; *p* = 0.005). AEs were reported in 61% of vaccinated patients after the first dose and in 40.5% after the second dose (*p* = 0.01). The most common reported AEs were pain at the injection site (54.5%), fever (22%), fatigue (21%), myalgia (10.5%), and headache (10%). A lower percentage of AEs was reported in older patients (OR = 0.3; *p* = 0.001). The COVID-19 vaccines are safe for PAH/CTEPH patients. The results obtained in this study may encourage patients of these rare but severe cardio-pulmonary diseases to get vaccinated against COVID-19.

## 1. Introduction

Pulmonary arterial hypertension (PAH) and chronic thromboembolic pulmonary hypertension (CTEPH) are rare severe diseases that lead to progressive right heart failure and death if left untreated. PAH (group 1 of clinical classification of pulmonary hypertension) is characterized by precapillary pulmonary hypertension and elevated vascular resistance on right heart catheterization [1,2]. PAH patients receive specific drugs that decrease their pulmonary arterial pressure and may reverse right heart remodeling and slow down progression of the disease. Chronic thromboembolic pulmonary hypertension (group 4 of clinical classification of pulmonary hypertension) is characterized by the presence of thromboembolic material in pulmonary arteries. Chronic obstruction of the pulmonary arteries leads to increased pulmonary arterial pressure and development of precapillary pulmonary hypertension [1]. Sometimes, CTEPH is preceded by an acute episode of pulmonary embolism [3,4]. All CTEPH patients require indefinite anticoagulation due to the risk of progression of the disease or recurrence of venous thromboembolism. When thromboembolic material is located in the proximal pulmonary arteries, patients are treated by pulmonary endarterectomy, whereas when it is located in the distal pulmonary arteries, patients are treated by pulmonary balloon angioplasty. Some CTEPH patients may need additional specific drugs to slow down the progression of the disease [1,3,4].

PAH and CTEPH patients need frequent medical contact and regular follow-up visits to specialist centers to prevent deterioration and progression of the disease, and to exclude possible side effects of specific drugs [1,5].

The COVID-19 pandemic has modified our lives and healthcare systems strive to minimize the risks of COVID-19 infection [5,6]. The frequency of COVID-19 infection among PAH and CTEPH patients is similar to that in the general population, but the severity and prognosis of the disease are much worse in these patients, with a mortality rate of around 12% in the USA and Europe [7,8]. For this reason and due to the rapid spread of the infection globally, COVID-19 vaccination, which is recommended by the World Health Organization, is crucial in preventing COVID-19 infection in the general population and in patients with PAH /CTEPH.

We present a multicenter cross-sectional study performed in Poland. The aim of our study was to identify reasons why PAH/CTEPH patients refused to be vaccinated against and to assess the safety profile of approved COVID-19 vaccines in vaccinated PAH/CTEPH patients. Moreover, we tried to identify the clinical features associated with negative decisions about COVID-19 vaccination and the impact of these clinical features on post-vaccination adverse events in PAH/CTEPH patients.

## 2. Materials and Methods

### 2.1. Study Group

This prospective observational study was performed in three pulmonary hypertension centers in Poland. Consecutive patients with PAH and CTEPH who agreed to participate in the study were enrolled. The inclusion criteria for the study were over 18 years of age and diagnosis of PAH or CTEPH confirmed by right heart catheterization and other necessary tests according to the current guidelines [1]. All the patients gave their written consent to participate in the study. The study protocol was approved by the Bioethics Committee of the Centre of Postgraduate Medical Education in accordance with the Declaration of Helsinki (number KBE 23/2021, date of approval: 14 April 2021).

### 2.2. Methods

The first assessment was conducted between April and mid-June 2021, when, on routine visit to the centers, patients were asked if they had received the COVID-19 vaccines. Additionally, they were asked to complete the Fear of COVID-19 scale (FCV-19S) and the Hospital Anxiety and Depression Scale (HADS). The FCV-19S questionnaire was chosen to assess the patients’ mental status and level of fear of COVID-19 during the pandemic [9]. It is a psychometric tool that consists of 7 items. Answers are given on a scale of 1 (strongly disagree) to 5 (strongly agree). Each patient chose from 1 to 5 for each statement and could get a total score of 7–35 points [9]. The higher the points, the higher the fear of COVID-19. The FCV-19S is widely used in clinics, and it has been translated into various languages, including Polish, and validated in various countries, including Poland [10,11]. The Hospital Anxiety and Depression Scale reflects the level of anxiety and depression. It consists of 16 items divided into anxiety (7 questions), depression (7 questions), and ratty (2 questions) subscales. Each item has four possible answers, and 0–21 points can be obtained for each subscale of anxiety or depression [12]. A cut-off value of 8 or more in the HADS anxiety part (HADS-A) or HADS depression part (HADS-D) can be used to determine patients who are anxious or depressed [13].

The obtained results about the mental status of PAH/CTEPH patients during the pandemic have been described in another paper [14]. In the beginning, we wanted to assess the mental status of patients during the pandemic. When we collected the earlier data and found that only 43% of PAH/CTEPH patients were vaccinated by mid-June 2021, we decided to investigate the reasons for the reluctance to be vaccinated in the study group. We also collected information about adverse events (AEs) of the COVID-19 vaccines. We conducted interviews with all PAH/CTEPH patients. Toward the end of September 2021, all the patients were asked again if they had received COVID-19 vaccination. We asked those who did not agree to be vaccinated their main reasons for such a decision. For patients that agreed to be vaccinated, we asked the type of vaccine they chose, the number of doses taken, and if there were side effects after the first and second doses. All vaccinated patients were immunized according to the recommended schedule time of the given vaccines. We started collecting data on 14 April 2021, hence the study group did not include patients who had died in our 3 centers due to COVID-19 infection (33 patients with PAH/CTEPH).

Information about the demographic characteristics of the patients, their clinical conditions and treatment were obtained from their medical records.

### 2.3. Statistical Analysis

Statistical analysis was performed using the Statistica software version 13.3 (TIBCO Software Inc., license acquired from the local distributor StatSoft Polska). Data distribution was tested using the Kolmogorov–Smirnov test. Categorical variables were presented as numbers and percentages; continuous variables were presented as medians and interquartile ranges, or means and standard deviations. For group comparisons, the Chi-square test, Fisher’s exact test, paired *t*-test, and Mann–Whitney U test were used as appropriate. Univariate and multivariate regression analyses were performed to identify factors associated with unwillingness to be vaccinated against COVID-19 and to determine factors that increased the risk of side effects after vaccination. Statistical differences were considered significant for *p* ≤ 0.05.

## 3. Results

### 3.1. Study Group

A total of 261 PAH/CTEPH patients were enrolled in the study.

The median age of participants was 60 years (range 18–92). Most of the patients were female (163, 62%). The study group consisted of 164 (63%) PAH and 97 (37%) CTEPH patients. Two hundred patients (77%) were vaccinated against COVID-19, while 61 patients (23%) did not agree to be vaccinated. In the vaccinated group, 125 patients (63%) had PAH and 75 (37%) had CTEPH, while in the unvaccinated group, 39 patients (64%) had PAH and 22 (36%) had CTEPH. For the PAH patients, 125 (76%) were vaccinated and 39 (24%) were unvaccinated. For the CTEPH patients, 75 (77%) were vaccinated and 22 (23%) were unvaccinated.

The vaccinated and unvaccinated groups differed in age. The vaccinated group was older than the unvaccinated group (median age 62.5 (18–92) vs. 52.3 (23–87), *p* = 0.005). There was no significant difference in the proportion of female patients between both groups (126 (63%) in vaccinated and 37 (61%) in unvaccinated). Vaccinated PAH/CTEPH patients had higher fear of COVID-19 than the unvaccinated patients (19 (7–35) vs. 16 (7–35), *p* = 0.04). There were no differences in other clinical factors (WHO functional class, duration of the disease, number of specific drugs used, number of concomitant diseases, history of COVID-19) between the vaccinated and unvaccinated groups. The anxiety (HADS-A) and depression components (HADS-D) of the Hospital Anxiety and Depression Scale did not differ between both groups. The baseline characteristics of the study group and subgroups are presented in Table 1.

### 3.2. Reasons and Factors Associated with Unwillingness to Vaccinate against COVID-19 in PAH/CTEPH Patients

**Figure 1:**
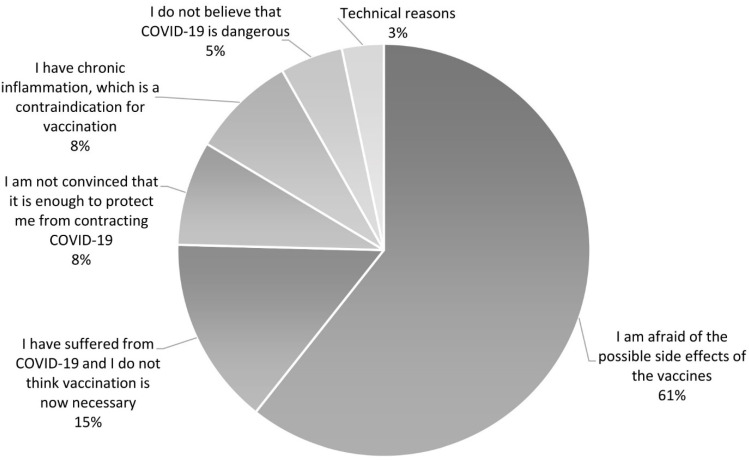
Reasons for the unwillingness of PAH/CTEPH patients to vaccinate against COVID-19.

Sixty-one PAH/CTEPH patients (23%) refused to be vaccinated against COVID-19. Unvaccinated PAH/CTEPH patients were asked the question: Why did you not get vaccinated? We divided the obtained responses into 6 main reasons. The main reason for refusing to vaccinate against COVID-19 was fear of the side effects of the vaccines (*n* = 37; 61% of unvaccinated patients). The second reason was the opinion that a history of COVID-19 infection is sufficient to protect against the next incidence of the disease and, thus, vaccination is unnecessary in those who have been previously infected (*n* = 9; 15% of patients). Third, patients were not convinced that vaccination would sufficiently protect them against COVID-19 (*n* = 5; 8%). Some patients did not believe in the existence of the SARS-CoV-2 virus, nor in the fact that the virus is dangerous (*n* = 5; 8%). Three (5%) patients who suffered from chronic inflammatory disease (1 patient had well-controlled ulcerative colitis, 1 had dermatitis of the lower legs, and 1 had elevated C-reactive protein after bacterial sepsis) reported that it is a contraindication for vaccination. Another 2 patients did not vaccinate due to organizational problems (1 could not go to the vaccination center and 1 did not have enough time to register for vaccination). The reasons why patients refused vaccinations are presented in Figure 1.

We tried to identify clinical and demographic factors that might have impacted decisions to take or not take the COVID-19 vaccines. In the univariate regression logistic analysis, elderly age ≥60 years (OR 2.7; 95%CI (1.5–4.9), *p* = 0.0015) and fear of COVID-19 in FCV-19S higher than the median increased the odds ratio of willingness to vaccinate against COVID-19 (OR 1.8; 95%CI (1.0–3.3), *p* = 0.049). In the multivariate regression analysis, only age greater than or equal to 60 years increased willingness to vaccine against COVID-19 in PAH/CTEPH patients (OR 2.5, 95%CI (1.3–4.6), *p* = 0.005). Results of the regression analyses are presented in Table 2.

### 3.3. Adverse Events after COVID-19 Vaccination in PAH and CTEPH Patients

**Figure 2:**
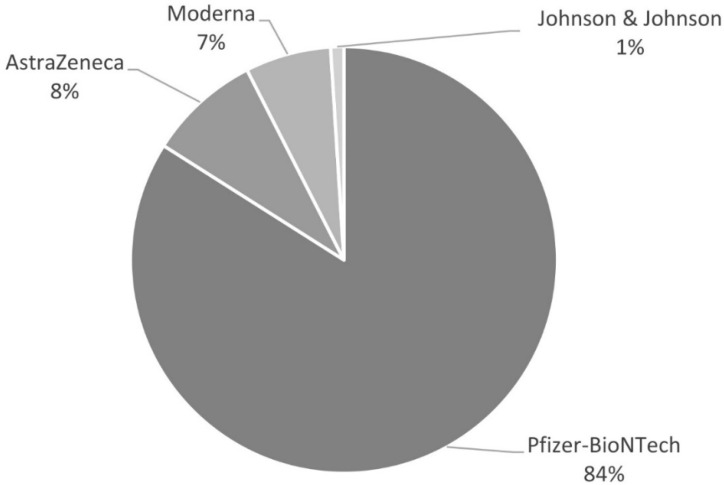
Types of COVID-19 vaccines received by vaccinated PAH/CTEPH patients.

Two hundred PAH/CTEPH patients (77%) were vaccinated against COVID-19. Most of the vaccinated patients received the Pfizer-BioNTech vaccine (84%), while the rest received the AstraZeneca, Moderna, and Johnson and Johnson vaccines (8%, 7%, and 2%, respectively). One patient received 1 dose of Pfizer-BioNTech and 1 dose of Moderna. The types of vaccines received by patients are presented in Figure 2.

**Figure 3:**
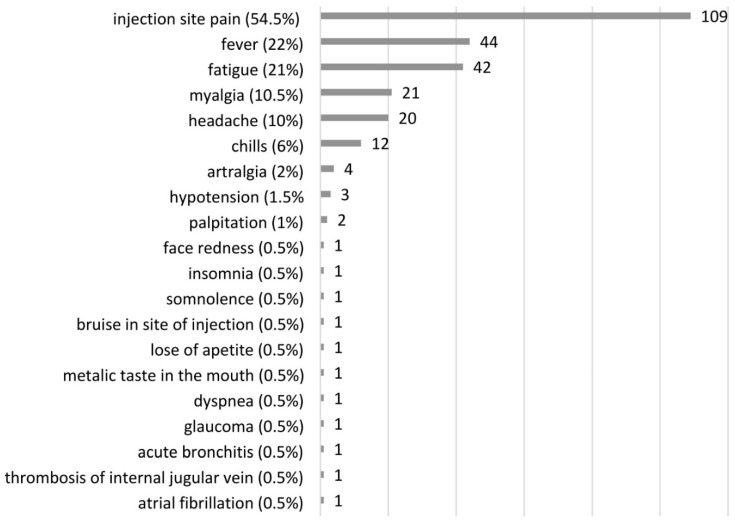
Side effects reported by PAH/CTEPH patients after COVID-19 vaccination.

Seventy-seven percent of vaccinated patients reported at least one post-vaccination adverse event (AE). The majority of AEs were mild or moderate and disappeared in a few days. The most frequently reported side effects were site injection pain (54.5%), fever (22%), fatigue (21%), myalgia (10.5%), headache (10%), and chills (6%). Serious adverse events (SAE) were reported by 5 patients. One patient with CTEPH was diagnosed with acute glaucoma on the third day after receiving the first dose of the AstraZeneca vaccine, which led to his refusal to take the second dose of the vaccine. One CTEPH patient, who had a rapid progression of bladder cancer with distal metastasis, had prolonged hypotension after receiving the second dose of the Pfizer-BioNTech vaccine, and thus needed to reduce his use of riociguat. One patient with a permanent cannula in their right subclavian vein and superior vena cava for continuous infusion of epoprostenol had venous thrombosis in their right internal jugular vein at the bend of the Hickman’s catheter. This episode was asymptomatic and detected accidentally on the seventh day after the second dose of the Pfizer-BioNTech vaccine had been administered, when the patient had their routine right heart catheterization to check if the treatment of the PAH disease was sufficient. This patient was taking apixaban due to their previous history of acute pulmonary embolism. One patient with CTEPH had acute bronchitis on the third day after receiving the second dose of the Pfizer-BioNTech vaccine, leading to their hospitalization. One patient with PAH had paroxysmal atrial fibrillation on the fifth day after receiving the first dose of the Pfizer-BioNTech, and electrical cardioversion was needed; there was no arrythmia after the second dose. One SAE was classified as probably related to the given COVID-19 vaccine and 4 SAEs as possibly related to the given vaccines. All reported adverse events in PAH/CTEPH patients are presented in Figure 3.

Thirty-three percent of the vaccinated patients had no side effects after receiving both the first and second doses of the COVID-19 vaccines. Side effects in vaccinated patients were more common after the first than the second doses (61% vs. 40.5% respectively, *p* = 0.01). The most frequently reported adverse events after the first doses were pain at the site of injection (50.5%), fever (17.5%), fatigue (14.5%), myalgia (8%), chills (8%), and headache (6%). The most common AEs after administration of the second doses of the vaccines were pain at the site of injection (28.5%), fever (10.5%), fatigue (13%), myalgia (6%), headache (6%) and chills (4%). The most frequent side effects after the first and second doses of vaccination against COVID-19 in PAH/CTEPH patients are presented in Table 3.

In the post-vaccination period, the patients frequently reported one or two AEs. The most common were pain at the site of injection and fever. Three or four sides effects in one patient were less frequent. The number of side effects after the first and second doses is presented in Table 4.

In the univariate regression logistic analysis, age greater than or equal to 60 years (OR 0.2; 95%CI (0.1–0.5), *p* = 0.0000), more advanced PAH/CTEPH disease, WHO functional classes 3 and 4 (OR 0.5; 95%CI (0.2–0.9), *p* = 0,03), and presence of concomitant disease (OR 0.4; 95%CI (0.2–0.8), *p* = 0.02) all decreased the frequency of reported AEs in the post-vaccination period. In the multivariate regression analysis, only age greater than or equal to 60 years decreased the frequency of reported AEs after COVID-19 vaccination (OR 0.3; 95%CI (0.1–0.5), *p* = 0.001). Results are presented in Table 5.

Forty five (17%) PAH/CTEPH patients had previously been infected with SARS-CoV-2. These patients were either unwilling to get vaccinated (*n* = 43) or received only 1 dose of the COVID-19 vaccine (*n* = 2). Nobody from the study group died due to SARS-CoV-2 infection since 14 April 2021, when we started collecting the data. No patient who received full vaccination against COVID-19, in accordance with the characteristics of the given type of vaccine, develop COVID-19 during the months of observation.

## 4. Discussion

PAH/CTEPH patients have an increased risk of death due to COVID-19 infection. The cumulative incidence of COVID-19 in individuals with PAH/CTEPH in the USA is 2.9 cases per 1,000 patients, a statistic similar to that of the general U.S. population. But the prognosis of COVID-19 in PAH/CTEPH patients is worse than in the general population [7]. Thirty percent of PAH/CTEPH patients with COVID-19 were hospitalized and 12% died [7]. In addition to the potential direct harm caused by COVID-19 during the pandemic, patients with PAH/CTEPH may have fewer visits in specialist centers, which may impact on the disease progression [7,15]. Similar data were described in a multicenter observational study in which most of the PAH/CTEPH patients were from Europe. The mortality for PAH and CTEPH patients altogether was 19%; it was 20 for PAH patients only and 14% for CTEPH patients only [8].

Experts strongly recommend that patients with chronic heart failure, including PAH/CTEPH patients should receive COVID-19 vaccination as soon as possible [16,17]. Other authors recommend prioritization of COVID-19 vaccination in patients with cardiovascular disease, according to the stage of the main disease and comorbidities. They propose that PAH patients classified under the WHO functional class 3 or 4 should be among the first segment of the population to be considered for COVID-19 vaccination [18].

Poland is one of the most populous countries in the European Union. The COVID-19 vaccination program in Poland started on 27 December 2020, when the first set of medical staff received the vaccines. At first, only healthcare and social workers could receive vaccination. On 25 January 2021, the vaccination program was launched for the oldest inhabitants aged over 80 years, with younger adults gradually incorporated [19]. From 10 May 2021, all Polish adult inhabitants could register for vaccination against COVID-19 [20]. Any PAH/CTEPH patients that wished to be vaccinated had at least 4 months and a half to do so.

Despite the recommendations, only 43% of patients were vaccinated by the middle of April 2021. After the benefits of the vaccination had been explained to the PAH/CTEPH patients, 77% of them were vaccinated by the end of September 2021. Although this was much higher than in the general population in Poland, where the full vaccination rate was 51.3% by the end of September 2021 [21], willingness to be vaccinated against COVID-19 in PAH/CTEPH patients was still insufficient. 

To our knowledge, there has been no specific study on the reasons for unwillingness to be vaccinated against COVID-19 among patients with PAH/CTEPH. Data about willingness to take the COVID-19 vaccines by patients with heart failure are sparse. Moderate acceptance levels of COVID-19 vaccination were observed among patients with severe heart disease who needed heart transplant. In one German transplant center, 74% of heart transplant recipients, 72% of patients on the heart transplant waiting list, and 56% of patients supported by left ventricular assist devices were vaccinated against COVID-19 [22]. A similar situation was observed in an oncological population in Poland. In mid-February 2021, 60.3% of patients were willing to get vaccinated against COVID-19, 16.2% were undecided, and 23.5% were unwilling [23].

The most common reason for unwillingness to vaccinate against COVID-19 in our PAH/CTEPH patients was fear of adverse events after vaccination, which was declared by 61% of patients. In December 2020 and March 2021, 63% and 65%, respectively, of the general Polish population who did not want to be vaccinated cited post-vaccination side effects as their reason [24]. Fifteen percent of our patients were convinced that their history of SARS-CoV-2 infection protected them sufficiently against reinfection. According to some observational studies, no reinfections were identified in a cohort of previously infected healthcare workers for approximately seven months after the first wave of the pandemic, but reinfections were seen after eight months [25,26]. At least 5 out of 9 PAH/CTEPH (55.5%) patients who felt that a history of SARS-CoV-2 infection protected them from reinfection had contracted the virus more than 8 months earlier. Compared to the general population in Poland, fewer percentage of people in our study declared that the pandemic did not exist or was a conspiracy (5% vs. 29% in the general population) and that they did not believe that vaccination was sufficient to protect them against COVID-19 (8% vs. 45%) [24].

The vaccinated and unvaccinated PAH/CTEPH groups differed in age (there were more elderly patients in the vaccinated group) and their levels of fear of COVID-19. Fear of COVID-19 in the study group was high, with a median score of 19 (7–35), and higher in the vaccinated group than in the unvaccinated group (19 (7–35) vs. 16 (7–35), *p* = 0.04). Fear of COVID-19 in our study group was as high as in cancer patients in Poland [27], and higher than in the general population in Poland [10].

In the univariate analysis, fear of COVID-19 greater than the median was associated with 1.8 times greater odds ratio of willingness to vaccinate (*p* = 0.049). In the multivariate regression analysis, only age greater than or equal to 60 years increased the odds ratio of willingness to vaccinate against COVID-19 in PAH/CTEPH patients (*p* = 0.005). The impact of advanced age in the study group on the decision to vaccinate against COVID-19 was so strong that it masked the effect of fear of COVID-19 on decision making in a multivariate analysis. Other clinical and demographic factors had no impact on the willingness to vaccinate against COVID-19. Another study identified age between 60–74 years as a factor that decreased the odds ratio of lack of willingness to vaccinate against COVID-19 in the general population in Poland [28], a result that is similar to ours. In our study, gender had no impact on the decision to vaccinate in PAH/CTEPH patients. This is different in the general population, with one paper finding that the female gender had a negative impact on willingness to vaccinate in Poland [28], while another paper found that women were more willing to be vaccinated and, in fact, more female are vaccinated in Poland [24]. There were higher odds of refusing COVID-19 vaccination in the rural areas, where the majority of people had no higher education, had more than three children, and had no access to the internet [28]. We did not analyze the socioeconomic impacts on willingness to vaccinate against COVID-19.

There are four vaccines authorized in Europe, all of which are used in Poland [20]. Most PAH/CTEPH patients (*n* = 168, 84%) in the vaccinated opted for Pfizer-BioNTech, 17 (8%) chose AstraZeneca, 13 (7%) chose Moderna, and 2 (1%) chose Johnson and Johnson. The first and second doses of all four vaccines were well tolerated. The majority of reported AEs were of mild or moderate intensity. These AEs included site injection pain (54.5%), fever (22%), fatigue (21%), myalgia (10.5%), and headache (10%). The frequencies of these AEs were similar to those in the general population [29,30]. Reported AEs were more frequent after the first doses of the vaccines than after the second, like in the general population [29,30,31,32]. Only 5 vaccinated patients (2.5%) suffered from severe side effects: 1 (0.5%) after first and 4 (2.0%) after second doses. No one in our study was diagnosed with thrombocytopenia with unusual thrombosis, reported as a possible severe adverse event of the AstraZeneca vaccine [33]. One patient reported an SAE probably related to the Astra Zeneca vaccine. He had acute glaucoma few days after receiving the first dose of the AstraZeneca vaccine, but we have no precise information if he had central or branch retinal vein occlusion. He had no thrombocytopenia. One patient had right internal jugular vein thrombosis on the seventh day after COVID-19 vaccination. The patient had a permanent cannula in their right subclavian vein and superior vena cava. Catheter-related thrombosis is a relatively common complication of central venous catheter insertion, and this is unrelated to the vaccination [34]. One patient had atrial fibrillation. In one pilot study, atrial fibrillation was noted in at least 5% of people ≥ 65 years old in the general population who were vaccinated against COVID-19 and agreed to check their heart rhythm [35]. In the current study, one patient had hypotension, but he had been receiving several drugs as hypertensive agents and riociguat for CTEPH treatment. He lost weight due to cancer progression but he did not reduce the use of the drugs, although he had symptomatic hypotension before vaccination. One patient had acute bronchitis and developed bacterial pneumonia.

In the multivariate regression analysis, only age greater or equal than 60 years decreased the odds frequency of AEs after COVID-19 vaccination. This result is similar to that of another study on the general population, in which the occurrence of side effects after COVID-19 vaccination in an elderly group aged ≥55 years was less frequent [29,30,31]. In our study group, 48% of PAH/CTEPH patients were on anticoagulants, but these had no beneficial or harmful effect on the frequency of reported AEs. The type of pulmonary hypertension or severity of the disease had no impact on AEs.

This study has some limitation. First, the examined group was relatively small. We did not perform analysis of socioeconomic factors that might have had some impacts on the decisions of PAH/CTEPH patients to take the COVID-19 vaccines. Due to the small sample size, we did not perform a comparative analysis of the adverse events of individual vaccines.

## 5. Conclusions

The COVID-19 vaccines are safe for PAH/CTEPH patients. Due to the high risk of death associated with SARS-CoV-2 infection in PAH/CTEPH patients, COVID-19 vaccination in this group should be a high priority.

The most common reasons for refusing to be vaccinated, such as concerns about the side effects of the vaccines and the belief of acquisition of permanent immunity due to a history of SARS-CoV-2 infection, can be changed through a factual conversation between the doctor and the patient.

The safety profiles of the COVID-19 vaccines in patients with PAH and CTEPH could convince people with these rare but severe diseases to get vaccinated.

## Figures and Tables

**Table 1:** Characteristics of the study group patients according to their decisions about vaccination against COVID-19.

	Total Study Group *n* (%) or Mean (SD)	Vaccinated *n* (%) or Mean (SD)	Unvaccinated *n* (%) or Mean (SD)	*p*-Value Vaccinated vs. Unvaccinated
Number of patients	261 (100%)	200 (77%)	61 (23%)	
Age, years	60 (18–92)	62.5 (18–92)	52.3 (23–87)	0.005 *
Sex, female	163 (62%)	126 (63%)	37 (61%)	0.78
Duration of disease, years	7.3 ± 7.1	7.5 ± 7.1	6.3 ± 7.1	0.15
PAH patients	164 (63%)	125 (63%)	39 (64%)	0.86
Idiopathic PAH	85 (52%)	69 (55%)	24 (41%)	
Heritable PAH	5 (3%)	3 (2%)	2 (5%)	
PAH associated with CHD	36 (22%)	27 (22%)	9 (23%)	
PAH associated with CTD	30 (18%)	22 (18%)	8 (20%)	
PAH porto-pulmonary	6 (4%)	3 (2%)	3 (8%)	
Drug-induced PAH	1 (0.5%)	1 (1%)	0	
PAH associated with HIV	1 (0.5%)	0	1 (3%)	
PAH monotherapy	48 (29%)	38 (30%)	10 (26%)	0.76
PAH two drugs	59 (36%)	41 (33%)	18 (46%)	
PAH three drugs	57 (35%)	46 (37%)	11 (28%)	
CTEPH patients	97 (37%)	75 (37%)	22 (36%)	0.86
CTEPH-BPA	73 (75%)	57 (76%)	16 (73%)	0.82
CTEPH-PEA	22 (23%)	16 (21%)	6 (27%)	0.68
CTEPH monotherapy (riociguat or sildenafil)	75 (77%)	56 (75%)	19 (86%)	0.41
WHO functional class	2.4 ± 0.7	2.4 ± 0.7	2.4 ± 0.8	0.92
1	22 (8%)	15 (8%)	7 (11%)	
2	119 (46%)	94 (47%)	25 (41%)	
3	108 (41%)	83 (41%)	25 (41%)	
4	12 (5%)	8 (4%)	4 (7%)	
COVID-19 disease	44 (17%)	31 (16%)	13 (21%)	0.49
Anticoagulation	126 (48.3%)	99 (49.5%)	27 (44.3%)	0.54
Concomitant disease	165 (63%)	130 (65%)	57 (75%)	0.36
Arterial hypertension	117 (45%)	96 (48%)	40 (53%)	0.10
Diabetes	43 (16%)	35 (18%)	12 (16%)	0.60
COPD	23 (9%)	19 (10%)	9 (12%)	0.73
Coronary artery disease	44 (17%)	37 (19%)	10 (13%)	0.41
Neoplasm	29 (11%)	18 (9%)	10 (13%)	0.29
Obesity, BMI ≥ 30 kg/m^2^	70 (27%)	54 (27%)	23 (30%)	0.92
Fear of COVID-19	19 (7–35)	19 (7–35)	16 (7–35)	0.037 **
HADS-A ≥ 8	78 (30%)	61 (31%)	17 (28%)	0.70
HADS-D ≥ 8	51 (20%)	36 (18%)	15 (25%)	0.48

PAH—Pulmonary arterial hypertension, PAH-CHD—Pulmonary arterial hypertension related to congenital heart disease, IPAH—Idiopathic pulmonary arterial hypertension, PAH-CTD—Pulmonary arterial hypertension associated with connective tissue disease, PAH-porto-pulmonary—Pulmonary arterial hypertension associated with portal hypertension, HIV-human immunodeficiency virus, CTEPH—Chronic thromboembolic pulmonary hypertension, BPA—Balloon pulmonary angioplasty, PEA—Pulmonary endarterectomy, COPD—Chronic obstructive pulmonary disease, WHO—World Health Organization, HADS—Hospital Anxiety and Depression Scale, HADS-A—anxiety part, HADS-D—depression part; * Mann-Whitney U test, ** Mann-Whitney U test.

**Table 2:** Impact of demographic and clinical factors on the willingness to vaccinate against COVID-19 in PAH/CTEPH patients: results of the univariate and multivariate regression analyses.

	Univariate Analysis OR (95%CI)	*p*-Value (LR)	Multivariate Analysis OR (95%CI)	*p*-Value (Wald)
Age ≥ 60 years	2.7 (1.5–4.9)	0.0015 *	2.5 (1.3–4.6)	0.005 *
Female gender	1.1 (0.6–2.0)	0.7		
CTEPH	1.0 (0.6–1.9)	0.8		
WHO functional class 3–4	0.9 (0.5–1.6)	0.7		
History of COVID-19	0.7 (0.3–1.4)	0.3		
Presence of concomitant disease	1.4 (0.8–2.5)	0.3		
Fear of COVID-19 ≥ median	1.8 (1.0–3.3)	0.049 *	1.7 (0.9–3.1)	0.09
HADS-A ≥8	1.1 (0.6–2.2)	0.6		
HADS-D ≥8	0.7 (0.4–1.4)	0.3		

CTEPH—chronic thromboembolic pulmonary hypertension, WHO—World Health Organization, HADS—Hospital Anxiety and Depression Scale, HADS-A—anxiety part, HADS-D—depression part; * *p* < 0.05.

**Table 3:** The most frequent side effects reported by PAH/CTEPH patients after the first and second doses of vaccination against COVID-19.

AEs	After First Dose (*n* = 200) *n* (%)	After Second Dose (*n* = 197) *n* (%)
Pain at the site of injection	101 (50.5)	56 (28.5)
fever	35 (17.5)	21 (10.5)
fatique	29 (14.5)	26 (13)
myalgia	16 (8)	12 (6)
chills	16 (8)	8 (4)
headache	12 (6)	11 (6)
other	11 (5.5)	10 (5)

PAH—pulmonary arterial hypertension, CTEPH—chronic thromboembolic pulmonary hypertension, AE—adverse event.

**Table 4:** Number of side effects in PAH/CTEPH patients after the first and second doses of vaccination against COVID-19.

Number of Side Effects	After First Dose (*n* = 200) *n* (%)	After Second Dose (*n* = 197) *n* (%)	*p*-Value
0	78 (39)	119 (59.5)	0.001 *
1	73 (36.5)	43 (21.5)	
2	24 (12)	22 (11)	
3	13 (6.5)	10 (5)	
4	9 (4.5)	4 (2)	
≥5	3 (1.5)	2 (1)	

PAH—pulmonary arterial hypertension, CTEPH—chronic thromboembolic pulmonary hypertension; * Mann-Whitney U test.

**Table 5:** Impact of different demographic and clinial factors on the freqency of side effects in PAH/CTEPH patients: results of the univariate and multivariate regression analyses.

	Univariate Analysis OR (95%CI)	*p*-Value (LR)	Multivariate Analysis OR (95%CI)	*p*-Value (Wald)
Age ≥ 60 years	0.2 (0.1–0.5)	0.0000 *	0.3 (0.1–0.5)	0.001 *
Female gender	1.2 (0.7–2.2)	0.7		
CTEPH	0.6 (0.4–1.2)	0.2		
WHO functional classes 3 and 4	0.5 (0.2–0.9)	0.03 *	0.8 (0.4–1.5)	0.5
Presence of concomitant disease	0.4 (0.2–0.8)	0.02 *	0.7 (0.4–1.5)	0.4

CTEPH—chronic thromboembolic pulmonary hypertension, * *p* < 0.05.

## Data Availability

Data sharing is not applicable to this article.
